# Coxsackievirus B3 infects and disrupts human induced-pluripotent stem cell derived brain-like endothelial cells

**DOI:** 10.3389/fcimb.2023.1171275

**Published:** 2023-04-17

**Authors:** Julia Mamana, Gabrielle M. Humber, Eric R. Espinal, Soojung Seo, Nadine Vollmuth, Jon Sin, Brandon J. Kim

**Affiliations:** ^1^ Department of Biological Sciences, University of Alabama, Tuscaloosa, AL, United States; ^2^ Department of Microbiology, Heersink School of Medicine, University of Alabama at Birmingham, Birmingham, AL, United States; ^3^ Center for Convergent Biosciences and Medicine, University of Alabama, Tuscaloosa, AL, United States; ^4^ Alabama Life Research Institute, University of Alabama, Tuscaloosa, AL, United States

**Keywords:** brain, blood brain barrier, stem cells, virus, coxsackievirus B3

## Abstract

Coxsackievirus B3 (CVB3) is a significant human pathogen that is commonly found worldwide. CVB3 among other enteroviruses, are the leading causes of aseptic meningo-encephalitis which can be fatal especially in young children. How the virus gains access to the brain is poorly-understood, and the host-virus interactions that occur at the blood-brain barrier (BBB) is even less-characterized. The BBB is a highly specialized biological barrier consisting primarily of brain endothelial cells which possess unique barrier properties and facilitate the passage of nutrients into the brain while restricting access to toxins and pathogens including viruses. To determine the effects of CVB3 infection on the BBB, we utilized a model of human induced-pluripotent stem cell-derived brain-like endothelial cells (iBECs) to ascertain if CVB3 infection may alter barrier cell function and overall survival. In this study, we determined that these iBECs indeed are susceptible to CVB3 infection and release high titers of extracellular virus. We also determined that infected iBECs maintain high transendothelial electrical resistance (TEER) during early infection despite possessing high viral load. TEER progressively declines at later stages of infection. Interestingly, despite the high viral burden and TEER disruptions at later timepoints, infected iBEC monolayers remain intact, indicating a low degree of late-stage virally-mediated cell death, which may contribute to prolonged viral shedding. We had previously reported that CVB3 infections rely on the activation of transient receptor vanilloid potential 1 (TRPV1) and found that inhibiting TRPV1 activity with SB-366791 significantly limited CVB3 infection of HeLa cervical cancer cells. Similarly in this study, we observed that treating iBECs with SB-366791 significantly reduced CVB3 infection, which suggests that not only can this drug potentially limit viral entry into the brain, but also demonstrates that this infection model could be a valuable platform for testing antiviral treatments of neurotropic viruses. In all, our findings elucidate the unique effects of CVB3 infection on the BBB and shed light on potential mechanisms by which the virus can initiate infections in the brain.

## Introduction

Coxsackievirus B3 (CVB3) is a common human virus that is a member of the *Picornaviridae* family and *Enterovirus* genus. CVB3 infections are generally subclinical or may cause mild flu-like illness which is typically self-resolving. CVB3 exhibits a wide tropic range to a number of internal organs, and as such, this virus can sometimes cause severe systemic diseases including myocarditis and pancreatitis (Huber and Ramsingh, 2004; Tam, 2006). Additionally, CVB3 is neurotropic and is a leading cause of non-bacterial, aseptic meningo-encephalitis, which can be especially harmful in young children (Beale et al., 1956; Berlin et al., 1993; Rotbart, 2000). Despite this, little is known regarding how CVB3 gains access to the central nervous system. To enter the brain, blood-borne pathogens must interact with and penetrate the brain barriers that are comprised of the BBB and the choroid plexus or the arachnoid barrier (Doran et al., 2005; Schwerk et al., 2015; Derk et al., 2022). The BBB represents the vast majority of barrier surface area in the brain and is comprised of highly specialized brain endothelial cells (BECs) (Wong et al., 2013). BECs line the blood vessels of the brain and are characterized by incredibly complex and robust tight junctions (TJs), as well as a high abundance of efflux transporters compared to peripheral endothelial cells (Abbott et al., 2010). BECs also maintain low rates of endocytosis in order to prevent entry of pathogens and circulating toxins, however a number of neurotropic pathogens have been demonstrated to interact with BECs to penetrate the BBB (Doran et al., 2005; Chen and Li, 2021). For example, the virus responsible for causing COVID-19, SARS-CoV-2, has been shown to enter the brain by disrupting the basement membrane of brain blood vessels (Zhang et al., 2021). Interestingly, TJ integrity does not appear to be altered during infection (Zhang et al., 2021). It has been suggested that HIV-1 crosses the BBB *via* a “Trojan horse” strategy, wherein virally infected immune cells traverse the BBB paracellularly (Ivey et al., 2009).

In recent years induced pluripotent stem-cell derived brain-like endothelial cells (iBECs) have become an attractive model to examine mechanisms of host-pathogen interactions (Kim et al., 2019b). Due to the high barrier function, TJ expression and efflux transporters, iBECs offer the ability to interrogate BBB properties *in vitro*. We had previously demonstrated that Group B *Streptococcus* (GBS) and *Neisseria meningitidis* (Nm) interact with BECs and can readily be modeled with iBECs (Kim et al., 2017; Kim et al., 2019a; Martins Gomes et al., 2019; Endres et al., 2022; Espinal et al., 2022a). Notably, using iBECs we were able to observe TJ destruction by GBS coordinated with the TJ repressor Snail1, previously demonstrated to be upregulated in immortalized models and mouse models (Kim et al., 2015; Kim et al., 2017). In addition, previously described GBS mutants identified as less able to interact with BECs had their phenotypes recapitulated in iBEC models (Doran et al., 2005; Seo et al., 2013; Mu et al., 2014; Kim et al., 2017). We have recently discovered that in addition to disruption of TJs, GBS is able to disrupt efflux transport and modulate macropinocytosis in iBECs highlighting how pathogens are able to disrupt key BBB characteristics (Kim et al., 2019a; Espinal et al., 2022a). As with GBS, Nm has also been able to be modeled using iBECs representing a notable leap as Nm is a human-specific pathogen and lacks robust *in vivo* models (Martins Gomes et al., 2019). A key advantage using iBECs is that they remain sensitive to other cell types of the CNS such as astrocytes, neurons, and pericytes (Lippmann et al., 2012; Appelt-Menzel et al., 2017; Canfield et al., 2017; Canfield et al., 2019). Recent work using iBECs and Nm has developed a meningeal cerebral-spinal fluid barrier in co-culture with iBECs and leptomeningeal cells that can alter Nm-BEC interaction (Endres et al., 2022). In addition to bacterial pathogens, iBECs have been used to examine virus-BEC interactions with a number of viruses including Zika, various flaviviruses, and SARS-CoV2 (Alimonti et al., 2018; Cheng et al., 2022; Krasemann et al., 2022). Here we present iBECs as a model system for exploring mechanisms of CVB3 infection of the BBB.

As mentioned previously, CVB3 is a leading cause of viral meningo-encephalitis, yet strikingly little is known regarding how the virus affects and eventually crosses the BBB. In the present study, we interrogated virus-BBB interactions by utilizing a human induced pluripotent stem cell-derived BEC (iBECs) model of infection. This *in vitro* model has key advantages over other *in vitro* BBB models as it maintains high amounts of efflux, low rates of endocytosis, and a physiologically comparable transendothelial electrical resistance (TEER) (Lippmann et al., 2012; Helms et al., 2016; Stebbins et al., 2016; Canfield et al., 2019). We and others have recently utilized the iBEC model to characterize BBB dysfunction with a variety of bacterial and viral pathogens highlighting the utility of probing BEC characteristics (Kim et al., 2017; Alimonti et al., 2018; Kim et al., 2019a; Martins Gomes et al., 2019; Endres et al., 2020; Cheng et al., 2022; Endres et al., 2022; Espinal et al., 2022a; Espinal et al., 2022b; Krasemann et al., 2022). In the present study, we found that iBECs are indeed permissive to CVB3 infection wherein the virus replicates to a high titer. After three days of infection, infected cells showed clear aberrations in the distribution of the TJ protein Occludin. This coincided with a progressive decline in TEER. Interestingly, the iBEC monolayer remained intact, despite the high degree of infection and barrier leakiness. When we infected iBECs seeded in transwells set above layers of HeLa cells (permissive cells known to be highly susceptible to CVB3 infection), we found that these iBECs could protect HeLa cells from infection during very early timepoints of infection. However, the HeLa cells did eventually become infected prior to any drop-off in TEER. Taken together, these observations show that iBECs are indeed susceptible to CVB3 infection and eventually lose their barrier integrity. iBECs do not succumb to rampant cell death like typical permissive cells, and rather, produce high amounts of virus for a prolonged period of time. In addition, iBEC monolayers can temporarily form effective physical barriers that prevent viral passage. However, once these cells are infected, they appear to release infectious virus on either side of the monolayer, whether transported paracellularly or through bilateral viral shedding.

## Materials and methods

### Reagents

iPSC line IMR-90-C4 was obtained from WiCell (WI, USA). HeLa RW cells were a generous gift from Ralph Feuer (San Diego State University, CA, USA). Stem-Flex media (Thermo Fisher Cat# A3349401), DMEM/F12 (Thermo Fisher Cat# 11-330-057), Knockout Serum Replacement (Thermo Fisher KOSR, Cat# 10-828-028), B27 (Thermo Fisher Cat# 17-504-044), human endothelial cell serum free media (hESFM, Thermo Fisher Cat# 11-11-044), Glutamax (Thermo Fisher Cat# 35-050-061), non-essential amino acids (NEAA, Thermo Fisher Cat# 11-140-050), Matrigel (Thermo Fisher Cat# CB-40234), Accutase (Stem Cell Technologies Cat# A1110501), Versene (Thermo Fisher Cat #15040-066) were obtained from Thermo Fisher Scientific. Collagen IV and tissue culture plastic was purchased from VWR. Collagen IV (Sigma Cat# C5533), Fibronectin (Sigma Cat# F1141) was purchased from Sigma-Aldrich.

### Maintenance of iPSCs and generation of iBECs

iPSCs were maintained as previously described on Matrigel coated 6 well plates and passaged using Versene every 3-4 days ensuring that less than 80% confluency was maintained (Stebbins et al., 2016; Espinal et al., 2022b). Maintenance iPSCs had daily changes of Stem-Flex media. iBECs were differentiated using established protocols weekly as described. Briefly, iPSCs were removed from the maintenance plate using Accutase and counted in single cell suspension (Stebbins et al., 2016; Endres et al., 2020; Espinal et al., 2022b). iPSCs were seeded at a density of 10,000/cm^2^ as this has been shown to be an optimum density for IMR-90-C4 (Wilson et al., 2015). Undifferentiated iPSCs were expanded for 3 days in Stem-Flex medium with daily media changes and differentiation was initiated by switching to unconditioned medium (UM) (Stebbins et al., 2016; Endres et al., 2020; Espinal et al., 2022b). Daily changes of UM medium occurred for 6 days. Next, cells were given endothelial cell medium + 10µM retinoic acid (RA) + 20ng/ml basic fibroblast growth factor (bFGF) for 2 days. iBECs were purified onto collagen IV/Fibronectin/water coated plastic or transwells at a ratio of 4:1:45 for plates and 4:1:5 for transwell inserts at densities of 1x10^6^ cells/cm^2^ for transwells or 5x10^5^ cells/cm^2^ for tissue culture plastic as previously described (Stebbins et al., 2016; Espinal et al., 2022b). The following day, cells were changed into endothelial cell media without RA or bFGF (Stebbins et al., 2016; Endres et al., 2020; Espinal et al., 2022b). Analysis for expression of markers and infection studies were conducted the next day as with previous work conducted on iBECs and infection (Kim et al., 2017; Kim et al., 2019a; Martins Gomes et al., 2019; Endres et al., 2020; Endres et al., 2022; Espinal et al., 2022a; Espinal et al., 2022b).

### TEER measurements and infection of iBECs

iBECs were purified at a density of 1x10^6^ cells/cm^2^ on transwells (Corning 3460) as previously described (Stebbins et al., 2016; Espinal et al., 2022a; Espinal et al., 2022b). iBECs were treated with CVB3 or DMEM at either a multiplicity of infection (MOI) of 1 or 10 as indicated. TEER was measured daily (each 24h as shown) using a Millicell ERS-2 Voltohmmeter (Millipore).

### Generation of CVB3 stocks

The pMKS1 plasmid used to generate CVB3 stocks was a generous gift from Dr. Ralph Feuer (San Diego State University, CA, USA). CVB3 stocks were generated from the pMKS1 plasmid as previously described (Slifka et al., 2001). Briefly, a plasmid containing the backbone of the myocarditic Nancy H3 variant of CVB3 (pH3) was engineered to include a unique SfiI restriction site allowing for insertion of foreign DNA fragments. Gene sequences for eGFP and timer protein were amplified from plasmids with sequence-specific primers with flanking SfiI sequences. PCR products were cloned into pMKS1 to generate constructs for eGFP-CVB3 and timer-CVB3. These constructs were then linearized by digesting with ClaI restriction enzyme (New England BioLabs; R0197S) and subsequently used for *in vitro* transcription using the mMESSAGE mMACHINE kit (Thermo Fisher; AM1344). Viral transcripts were then transfected into HeLa RW cells using Lipofectamine 2000 (Thermo Fisher; 11668019). Once 50% of transfected cells expressed viral eGFP, cells were then scraped, subjected to three freeze-thaw cycles, and centrifuged at 2,000 RPM to remove cellular debris. The resultant supernatant was referred to as “passage 1 virus”. For viral expansion, “passage 1 virus” was then overlain onto a second set of HeLa cells which were similarly harvested once those cells exhibited 50% viral eGFP expression. This “passage 2 virus” was subsequently used for downstream experiments. Viral propagation did not exceed “passage 2”.

### Infection of iBECs with CVB3

Viral infections of iBECs were performed at the MOIs indicated per experiment. The initial number of iPSCs seeded was used to calculate MOIs. Cells were inoculated with frozen viral stock with viral load calculated through plaque assays. Mock infected cells received equivalent volumes of DMEM growth medium. Vehicle for all experiments unless stated otherwise is unconditioned DMEM in equal volume as virus used for appropriate MOI.

### Immunofluorescence

At the time points indicated, CVB3 infected cells were imaged using a Nikon Ti2 inverted epifluorescence microscope equipped with a Qi2 camera (Nikon, Tokyo, Japan) using NiS Elements software version AR.5.30.05. Cells were fixed in ice-cold methanol and stained with anti-PECAM-1 (CD-31) (Cat# PA5-16301 Thermo Fisher), anti-VE-Cadherin (Cat# sc-52752 [BV9] Santa Cruz), anti-Glut1 (Cat# MA5-11315 Thermo Fisher), anti-P-gp (Cat# MA5-13854 Thermo Fisher), anti-Occludin (Cat# OC-3F10, Invitrogen), anti-Claudin-5 (Cat# 35-2500, Invitrogen), and anti ZO-1 (Cat# 33-9100 Invitrogen) as previously described (Stebbins et al., 2016; Espinal et al., 2022b) and imaged on the Nikon Ti2 microscope. Images were analyzed with NIH ImageJ software (FIJI).

### Viability of infected iBECS with trypan blue

iBECs were infected with CVB3 at MOI 10 and were assessed for viability at 48 h post infection (PI), 72 h PI, 5 days PI, and 9 days PI. The monolayer was trypsinized (Thermo Fisher Cat # 25200056**)** and incubated for 10 minutes. Viability was assessed per microliter of the trypsin solution using a one-to-one ratio of trypan blue (Thermo Fisher Cat# 15250061). Appropriate volume of trypsin dilution with trypan blue was pipetted onto a Countess slide (Thermo Fisher Cat# 10314) where viable cells were counted using the Countess 3 Cell Counter (Fisher Scientific, MA, USA).

### Quantification of infectious virus

Infectious virus was quantified *via* viral plaque assay. Briefly, confluent monolayers of HeLa RW cells were seeded onto 6-well plates and subsequently overlain with 400 µl of serially-diluted infectious fluid. Following one hour (h) of viral adsorption, monolayers were then overlain with 4 ml mixture of 0.6% molten agarose (Fisher Scientific; 16500-500) and 1x DMEM (Thermo Fisher; 12100046). 48 h after this overlay, DMEM+agarose plugs were fixed with plaque fixative solution containing 75% methanol and 25% acetic acid. After 20 minutes of fixation, fixed plugs were removed using a fine point weighing spatula, and the remaining HeLa RW monolayer was counterstained with crystal violet solution contain 0.5% crystal violet and 20% ethanol. Following 1 h of staining, cell monolayers were then washed in running tap water, allowed to let dry, and plaques were subsequently counted to calculate plaque forming units per ml.

### Sample collection for plaque assays

For the 9-day study on PFU, samples were collected from media samples of wells in triplicate every 24 h up to 216 h PI. Replacement of media immediately upon removal of sample was conducted with unconditioned cell media. For the collection of samples from transwells for analysis of virus production by iBECs, samples were collected from both the top compartment and the lower compartment at 6 h and 24 h PI. Samples experienced a single freeze thaw before plaque assays were initially ran. The plaque assays conducted on the iBECs in the upper compartment at 24 h PI of the transwell were trypsinized (Thermo Fisher Cat # 25200056**)** and incubated for 10 minutes, before being diluted in an equal volume of DMEM with antibiotics and FBS, as this is the preference for HeLas. These samples were then subjected to 3 total freeze thaw cycles followed by disruption with a 27 gauge needle. Samples were then run by plaque assay.

### Protection assays of permissive cells

To model the barrier function of the BBB iBECs were seeded in a transwell above HeLas and then infected at an MOI of 10. Controls used were wells with blank transwells infected at MOI 10 and wells with no transwell infected directly in the media. DMEM growth media was used for this experiment. Images displayed are images of the HeLa cells in the bottom compartment of the well. A second experiment was conducted to quantify virus alone where there were no HeLas lining the bottom of the well. A blank transwell and iBEC lined transwell were infected at an MOI 10. Media was collected at 2 h intervals with replacement for plaque assays for 24 h. At 24h cells were collected for plaque assays.

### Immunoblotting

Whole lysate cell samples were harvested by first aspirating media from cells, washing once with phosphate-buffered saline, and disrupting cells in RIPA buffer containing protease inhibitor cocktail (Roche; 05056489001). Protein concentration was determined using a bicinchoninic acid solution kit (Sigma-Aldrich, B9643). Equal amounts of protein were loaded into 4-20% Tris-Glycine SDS PAGE gels (Life Technologies; EC6025) and transferred to nitrocellulose membranes (VWR; 27376-991). Following transfer, adequate transfer and equal protein loading was determined by staining with Ponceau S solution (Sigma-Aldrich; P7170). Membranes were then blocked in blocking solution comprised of 3% bovine serum albumin (Millipore Sigma; A7906) dissolved in tris-buffered saline with 0.1% Tween-20 (Millipore Sigma; P1379) (TBS-T) for one h at room temperature. Membranes were then incubated in anti-coxsackievirus B monoclonal antibody (Mediagnost; M47) diluted in blocking solution overnight at 4°C. Membranes were then washed three times in TBS-T followed by incubation in anti-mouse secondary (1:3000, Millipore Sigma; 12-349). Membranes were then washed three times in TBS-T and protein signal was detected by incubating membranes in chemiluminescence substrate (Fisher Scientific; PI32209) and then imaging using an iBright FL1500 gel imaging system (Fisher Scientific, MA, USA). Densitometric analysis was performed using FIJI software where Viral Capsid Protein 1 (VP1) intensity was normalized to background intensity of the western blot.

### Statistics

Statistical analyses were conducted using GraphPad Prism. For pair-wise comparison a students *t*-test was used to determine significance. For multiple comparisons ANOVA was used to determine significance. A *p* < 0.05 was accepted as statistical significance.

## Results

### Human induced pluripotent stem cell-derived brain-like endothelial cells are susceptible to coxsackievirus B infection

**Figure 1:**
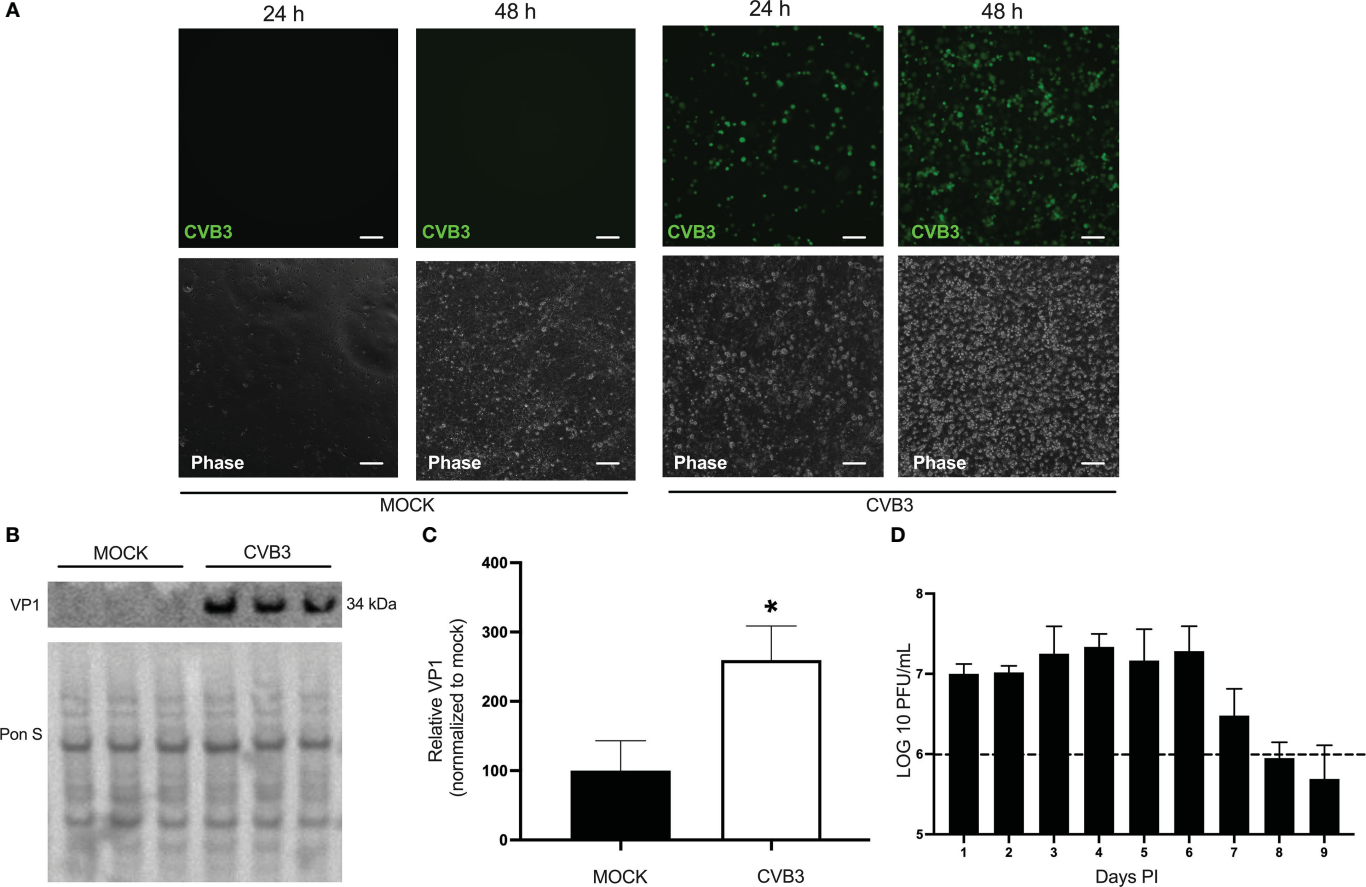
Human induced pluripotent stem cell-derived brain-like endothelial cells are susceptible to coxsackievirus B infection. iBECs were infected with eGFP-CVB3 or an equal volume of DMEM at MOI 10 for the durations indicated. **(A)** Fluorescence microscopy images (top) detecting viral eGFP. Phase contrast images of the same field are shown below. Scale bars= 100 µm. **(B)** Western blot detecting viral capsid protein VP1 in iBEC lysates that were either infected with eGFP-CVB3 at MOI 10 or mock infected with equivalent volume DMEM for 48 h PI. Ponceau S (Pon S) staining of the membrane is shown below. **(C)** Densitometric quantification of **(B)**. **(D)** Plaque assay quantification of infectious viral titers of media from iBECs infected with eGFP-CVB3 from tissue culture plastic coasted plates at MOI 10 (1.12x10^6^ PFU/mL) or mock treated with equivalent volume DMEM. Dotted line represents initial inoculum (*p<0.05; student’s *t*-test; n=3. Error bars represent standard deviation).

Coxsackievirus B (CVB3) is a neurotropic virus that is able to gain access to the brain and cause meningo-encephalitis in humans. It is unclear what type of cell-virus interactions occur at the BBB. We first sought to determine if iBECs were indeed susceptible to CVB3 infection. iBECs were generated using established protocols and confirmed by immunofluorescence for common BEC markers (**Supplemental Figure S1**). We infected monolayers of iBECs with CVB3 expressing enhanced green fluorescent protein (eGFP-CVB3) at MOI 10. Fluorescence microscopy on infected iBECs confirmed infection at MOI 10 as viral eGFP expression was apparent as early as 24 hours (h) post infection (PI) (**Figure 1A**). Western blots on cell lysates revealed the presence of intracellular viral capsid protein VP1 (**Figures 1B, C**), and plaque assays on cell media collected from wells daily show that infected cells were indeed releasing high titers of infectious virus up to 9 days (d) PI. (**Figure 1D**). Interestingly, iBEC viability at all time points was not significantly altered after CVB3 infection compared to mock treated, suggesting that iBECs may be resistant to viral-mediated cell death (**Supplemental Figure S2**) Taken together these data demonstrate that iBECs are indeed susceptible to infection and release high titers of infectious virus.

### CVB3 infected iBECs display signs of tight junction disruption

**Figure 2:**
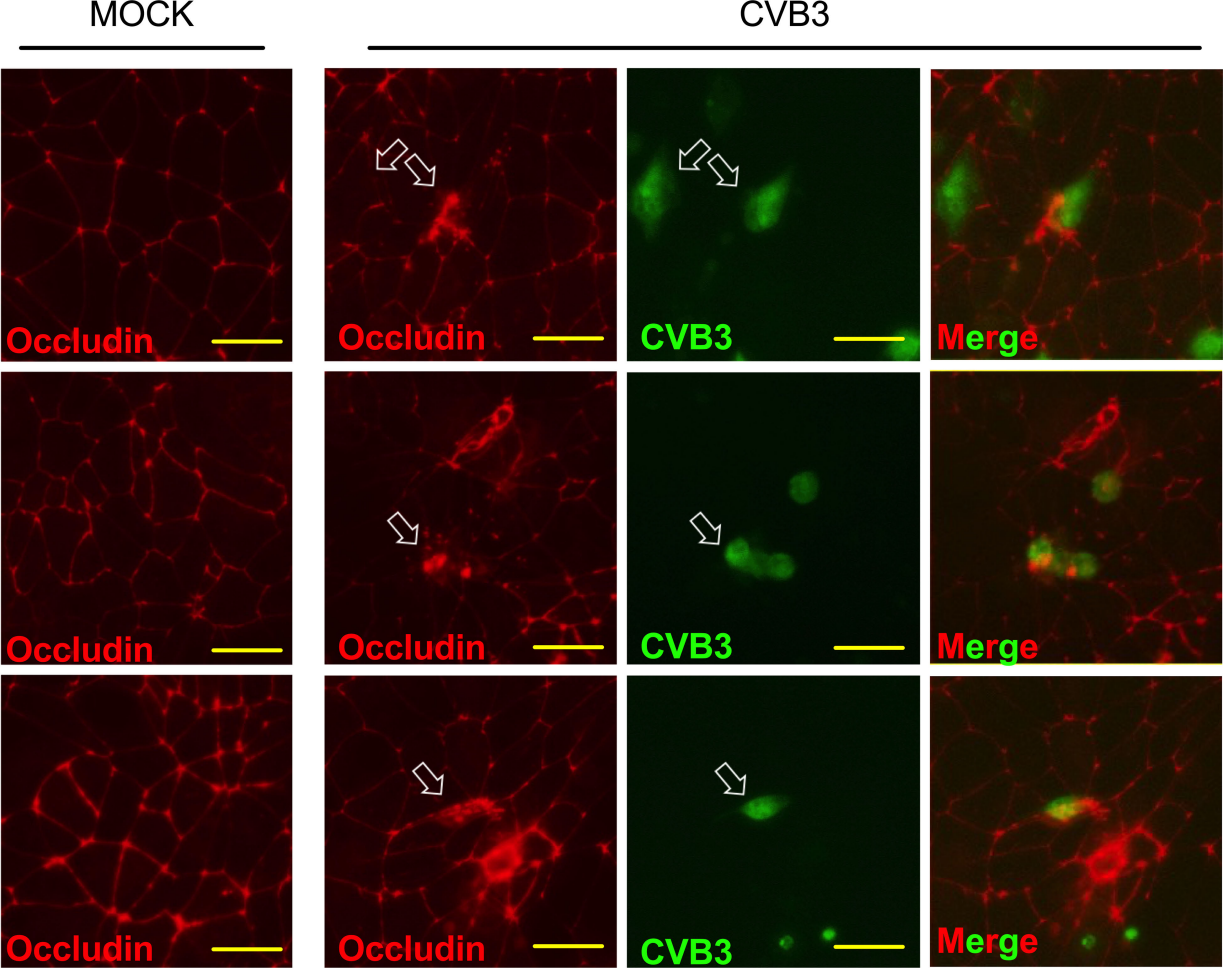
Infected iBECs display signs of tight junction disruption. Immunostaining detecting Occludin localization in iBECs either infected with eGFP-CVB3 at MOI 10 or mock treated with equivalent volume DMEM for 72 h. Empty white arrows indicate disrupted Occludin distribution. For eGFP-CVB infected cells, viral eGFP expression is shown to the right. Merged images are of infected cells with eGFP overlayed over Alexa Fluor 594 Occludin. Three representative images are shown for mock and CVB3 infected. Scale bars= 50 µm.

Examining infected iBECs showed that though these cells are susceptible to CVB3 infection, unlike typical permissive cell types such as HeLa, Vero, and HL-1 cells, iBECs do not display rampant amounts of cytopathic effect and rather maintain a continuous adherent monolayer (Robinson et al., 2014; Sin et al., 2017; Germano et al., 2019; Taylor et al., 2020). To determine if barrier properties might be altered following infection, we immunostained infected iBECs 72 h PI to detect TJ proteins Occludin, Claudin-5, and ZO-1. iBECs express these TJ proteins in a typical cobblestone pattern with continuous unbroken lines forming between adjacent cells. Interestingly, increasing delocalization of these proteins can be seen in a time dependent manner PI. At 72 h PI, Occludin appears to be relocalized from cell-cell junctions to intracellular puncta in infected cells (**Figure 2**). In addition, overall Occludin, Claudin-5, and ZO-1 staining intensity is markedly reduced in infected cells at later timepoints when compared to mock controls (**Supplemental Figure S3**). At 5 d PI, images revealed large areas of the infected iBEC monolayer with low or undetectable Occludin, Claudin-5, and ZO-1, despite a monolayer of adherent cells still present, indicating that the breakdown of TJs occurs with infection, potentially contributing to leakiness of the barrier (**Supplemental Figure S3**). These data suggest that CVB3 infection may contribute to dissipation of TJ protein localization in infected iBECs.

### CVB3 infection of iBECs leads to eventual loss of transendothelial electrical resistance

**Figure 3:**
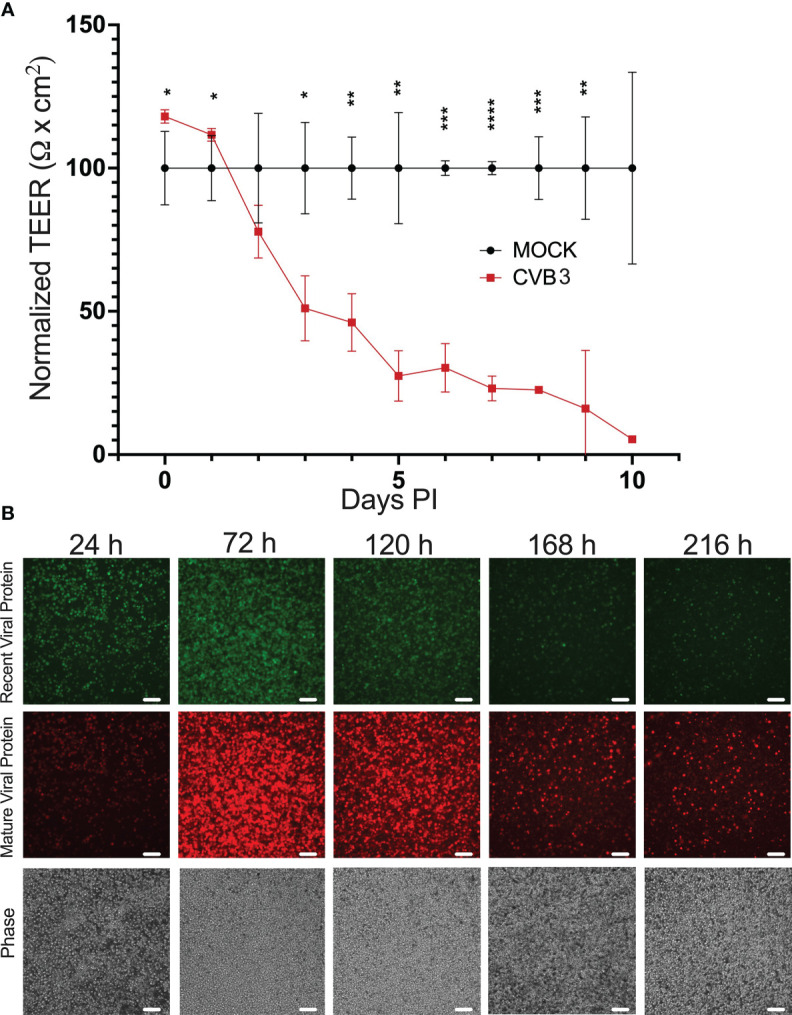
CVB3 infection of iBECs leads to eventual loss of transendothelial electrical resistance. iBECs were infected with CVB3 at MOI 10 for the durations indicated. **(A)** Transendothelial electrical resistance (TEER) in iBECs either infected with eGFP-CVB3 at MOI 10 or mock treated with equivalent volume DMEM. Relative TEER reading for virally-infected group was normalized to mock-infected group at each timepoint. **(B)** Fluorescence microscopy images of iBECs infected with Timer-CVB3 at MOI 10 for indicated timepoints. Green fluorescence, red fluorescence, and phase contrast panels are of the same field for each timepoint. Scale bars= 100 µm. (*p<0.05, **p<0.01, ***p<0.001, ****p<0.0001; student’s *t*-test; n=3. Error bars represent standard deviation).

Our previous immunostaining analysis revealed that CVB3-infected iBECs displayed aberrant TJ protein localization suggesting perturbation of TJs, thus we next assessed whether infection could alter overall barrier function by measuring transendothelial electrical resistance (TEER). To interrogate this, we infected iBECs with CVB3 expressing fluorescent Timer protein (Timer-CVB3) at MOI 10. Timer protein, previously developed by Terskikh et al, is a mutated dsRed that transitions slowly from green to red fluorescence over the course of approximately 24 h (Terskikh et al., 2000). Thus, cells recently infected with the Timer-CVB3 would fluoresce green, whereas cells that had been infected over 24 h prior would fluoresce red (Robinson et al., 2014). Following infection, we found that TEER in the infected cells remained comparable to mock-infected controls up to 48 h PI despite obviously high amounts of infection; however TEER values sharply declined at 3 days (d) PI and beyond (**Figure 3A**). This infection-mediated attenuation in TEER may represent an avenue by which circulating virus could gain easier access to the brain. Consistent with our earlier observations, TEER was almost non-existent at 7 d PI even though infected cells still formed an adherent iBEC monolayer (**Figure 3A**), suggesting that the loss of TEER may not be attributable to cell death. Additionally, in the infected group, red viral Timer protein continued to accumulate throughout this infection time course, while green Timer protein could also be observed up to 7 d PI, indicating new CVB3 infection was ongoing at these very late timepoints (**Figure 3B**). Plaque assays confirmed continued shedding of infectious virus until 9 d PI at both MOI 1 and MOI 10 (**Supplemental Figure S4**), which together with the viral Timer protein analysis, implicate that iBECs can support some degree of persistent CVB3 infection, but progressively lose barrier function at later timepoints. We observed a similar but much more gradual dissipation of TEER when infecting with MOI 1 (**Supplemental Figure S5**).

### Treatment with SB-366791 inhibits CVB3 infection in iBECs

**Figure 4:**
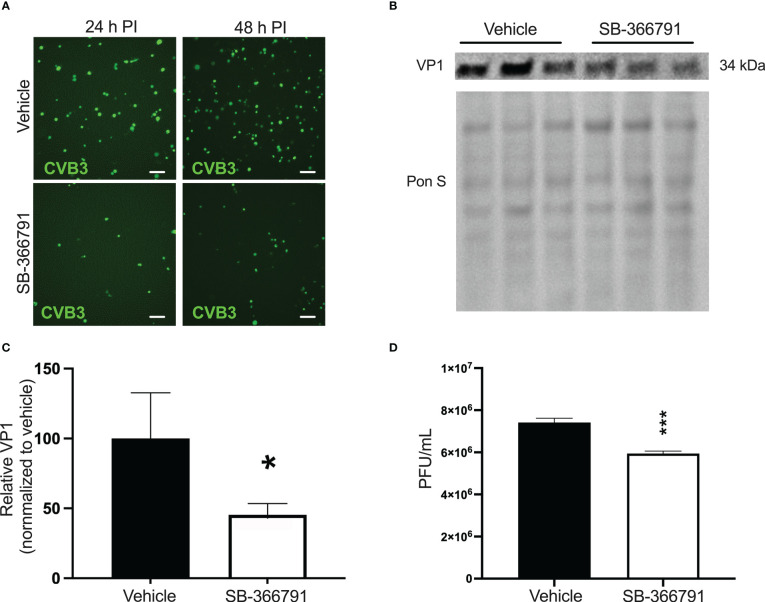
*Treatment with SB-366791 inhibits CVB3 infection in iBECs* iBECs were pretreated with 10 µM SB-366791 or DMSO (vehicle) for 24 h prior to infecting with eGFP-CVB3 at MOI 10. **(A)** Fluorescence microscopy images detecting viral eGFP at indicated timepoints. Scale bars= 100 µm. **(B)** Western blot detecting viral capsid protein VP1 in iBEC lysates from cells in **A** 48 **h** PI. Ponceau S staining of the membrane is shown below. **(C)** Densitometric quantification of **B** normalized to background intensity 48 h PI. **(D)** Plaque assay quantification of infectious viral titers of media from iBECs (*p<0.05, ***p<0.001; student’s *t*-test; n=3. Error bars represent standard deviation).

Recently, we had reported that CVB3 infection is supported by the heat/capsaicin sensor TRPV1 (Taylor et al., 2020). In that study, we found that treatment with the specific TRPV1 inhibitor SB-366791 significantly reduced CVB3 infection in HeLa cells (Taylor et al., 2020). Based on this, we tested if SB-366791 treatment could similarly suppress CVB3 infection in iBECs. We treated cells with 10 µM SB-366791 for 24 h prior to infecting with eGFP-CVB3 at MOI 10. Fluorescence microscopy revealed that SB-366791 treatment reduced viral eGFP expression at 48 h PI (**Figure 4A**). Western blots showed a significant reduction in intracellular VP1 levels 48 h PI (**Figures 4B, C**). Furthermore, plaque assays showed suppressed release of infectious virus in SB-366791 treated cells 48 h PI (**Figure 4D**). Quantity of live virus was modestly but significantly decreased after treatment with SB-366791, supporting the hypothesis that SB-3667791 is able to attenuate both virulence and viral replication. These data confirm that the antiviral effects of SB-366791 could be recapitulated in this iBEC model of CVB3 infection and suggest that this drug could be a promising intervention for the treatment of CVB3 brain infections.

### iBECs transiently protect permissive cells from infection

**Figure 5:**
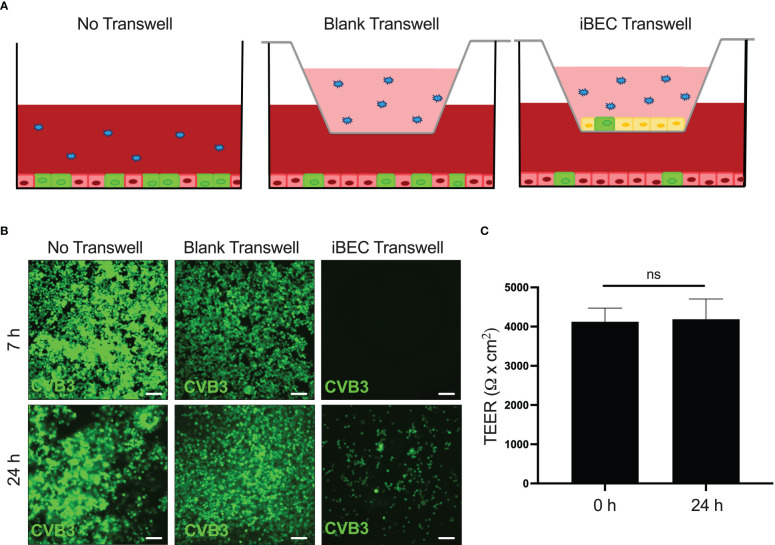
iBECs transiently protect permissive cells from infection iBECs were seeded in transwells and overlain above monolayer of permissive HeLa cervical cancer cells. iBECs were then infected with eGFP-CVB3 at MOI 10 with the viral inoculum added into the top of the transwell. **(A)** Schematic of experimental setup also detailing “No Transwell” and “Blank Transwell” control conditions. **(B)** Fluorescence microscopy images of HeLa cells (below transwells) detecting viral eGFP at indicated timepoints. Scale bars= 100 μm. **(C)** TEER measurements of iBEC-seeded transwells prior to infection and 24 h post-infection with eGFP-CVB3 at MOI 10. (ns, not significant, p>0.05; student’s t-test; n=3. Error bars represent standard deviation).

Our data up to this point demonstrate that CVB3 infects iBECs to high titer, and over the course of several days, TJs are disrupted and TEER declines. We next sought to determine if these aspects of compromised barrier function coincide with the ability for CVB3 to cross the iBEC monolayer. To test this, we seeded iBECs in a transwell insert and overlaid them over highly permissible HeLa cells. We then inoculated eGFP-CVB3 at MOI 10 above the transwell to determine if passage of the virus to the HeLa cells would be impeded by the iBECs **(** **Figure 5A** **)**. At 7 h PI, we found that iBEC-seeded transwells prevented infection of HeLa cells below, whereas HeLa cells below empty transwells already expressed viral eGFP indicating productive infection (**Figure 5B**). At 24 h PI, infected HeLa cells could be observed below iBEC transwells. However, consistent with our earlier observations, simultaneous TEER measurements revealed that at this 24 h infection timepoint, there was not yet any change in TEER (**Figure 5C**), suggesting that viral traversal of the iBEC monolayer was unlikely due to barrier leakiness, but rather was likely attributable to infected iBECs shedding virus into the HeLa cell compartment as iBEC infection was observed as early as 24 h PI. In all, our data reveals that though CVB3 could enhance BBB traversal by gradually dissipating integrity of the barrier, the virus may also be able to cross the BBB at early points of infection *via* basolateral viral shedding from infected BBB endothelial cells. To identify the rate at which virus enters the basolateral compartment of the transwell, we analyzed live virus in all compartments at 6 (**Supplemental Figure S6A**) and 24 h (**Supplemental Figure S6B** **).** We found the blank transwell readily had observable live virus in the basolateral compartment at both 6 and 24 h, however in transwells containing iBECs viral titers in the basolateral compartment was greatly attenuated at both timepoints (**Supplemental Figure S6** **)**. These data suggest that while iBECs may provide a robust physical barrier to CVB3, mechanisms of virus crossing whether paracellularly or bilateral shedding remain unexplored.

## Discussion

Our work investigates the ability for clinically relevant CVB3 to access the brain. Though CVB3-mediated neuroinflammatory illness is well-documented and the long-term consequences of such infections has been extensively described, very little literature exists on how the virus actually gains access to the brain, and surprisingly, there is a dearth of knowledge regarding what occurs when CVB3 interacts with the BBB. Previous studies have described infection of immortalized human cerebral microvascular cells (hCMECs) with an array of enteroviruses and showed infection of the brain endothelial barrier (Volle et al., 2015). Similar work has been conducted using primary human brain microvascular endothelial cells, providing relevant information about these viruses and the interaction with the BBB (Gu et al., 2023). To our knowledge, this is the first study to describe CVB3 infection of stem cell derived brain-like endothelial cells. Common *in vitro* BBB models typically revolved around the use of immortalized cell lines such as human brain microvascular endothelial cells (hBMECs) or hCMEC D3s which retain some aspects of the native BBB endothelium, but display reduced expression of TJ proteins and have relatively low TEER (Helms et al., 2016). *In vivo* modeling is another method that is sometimes used, and this has inherent advantages by capturing BBB dynamics in a fully-intact physiological state. However, dissecting BBB-related phenomena from the rest of the brain is challenging, and species variability would potentially limit translational value. To address these challenges, we chose to interrogate CVB3-BBB interactions using the established iBEC model, which is not only of human origin, but exhibits high efflux transporter expression, low endocytosis, and robust TJs that recapitulate TEER to a physiologically comparable level. While the iBEC model system offers strengths, it also has potential for improvement by incorporating other cells of the neurovascular unit such as neighboring astrocytes, neurons, and pericytes (Canfield et al., 2017; Canfield et al., 2019). Other brain barriers may contribute to pathogen access to the CNS such as the choroid plexus (CP) and the arachnoid barrier (Lauer et al., 2018; Derk et al., 2022). The CP is a highly vascularized region of the brain that is sealed from the CNS by the presence of CP epithelial cells. Interestingly the CP has been described as a potential CNS access point for a number of viral pathogens including CVB3 (Falangola et al., 1995; Feuer et al., 2003; Tabor-Godwin et al., 2010; Schneider et al., 2012; Dahm et al., 2017; Dahm et al., 2018; Lauer et al., 2018; Jacob et al., 2020; Kim et al., 2020; O’hara et al., 2020; Pellegrini et al., 2020; Wiatr et al., 2020). While, the arachnoid barrier and the CP have been shown to contribute to pathogen entry to the brain by viruses or CVB directly; the endothelial BBB also represents a large surface area in contact with the circulation that has been demonstrated to facilitate access to the CNS (Al-Obaidi et al., 2018; Calderón-Peláez et al., 2019; Mustafá et al., 2019; Kadry et al., 2020; Chen and Li, 2021; Erickson et al., 2021; Singh et al., 2021). The actual contribution of each barrier to CVB3 entry remains unknown, therefore, continued work identifying mechanisms BBB and CP dysfunction during infection remain relevant and an urgent need.

Using iBECs for our infection model, we were able to determine that these cells are indeed susceptible to CVB3 infection. Strikingly, we also observed that these cells harbor infection for unusually long periods of time (upwards of 7 days) and are resistant to virally-mediated cell death compared to typical CVB3-permissive cells. Because CVB3 has classically been thought to induce acute infection and cell lysis, it is somewhat unusual that iBECs could remain infected for so long, shed large amounts of virus, yet still maintain a continuous adherent cellular monolayer even at late infection timepoints. This aspect could be reconciled by work we and others had previously done showing that a number of enteroviruses (including CVB3) could escape the infected cell *via* released envelope-like microvesicles (Robinson et al., 2014; Chen et al., 2015; Sin et al., 2017; Huang et al., 2020; Netanyah et al., 2020). This non-lytic mode of viral egress allows for viral release without the necessity of host cell death, thus prolonging viral replication. It is feasible that iBECs could support vesicle-based viral egress, thus highlighting a potentially important viral mechanism that needs to be further explored in this model. It is interesting that this inherently robust nature of the BBB endothelium may represent a double-edged sword, as complete virally-mediated destruction of the BBB would of course have massively detrimental consequences, but the heartiness of the BECs could make them an ideal host cell type for prolonged productive viral infections. Another potential hypothesis as to why CVB3 exhibits such unusually long infection times with limited death could be explained by persistent infection. CVB3 was observed to exhibit persistent infection, especially in physiologically presenting chronic myocarditis and dilated cardiomyopathy (Chapman and Kim, 2008). In these disease contexts, CVB3 persisted beyond the acute phase to the point of non-productive infection (Chapman and Kim, 2008). Though CVB3 is responsible for a large portion of aseptic meningo-encephalitis cases, it would be interesting to interrogate virus-BBB interactions among other neurotropic viruses using the iBEC model. Echoviruses comprise the largest category of enteroviruses and are known for causing a wide range of human disease pathologies across Europe (Carrol et al., 2006; Benschop et al., 2021). With over thirty distinct strains, echoviruses are a diverse viral subcategory that contribute to similar pathologies as CVB3 including viral meningitis (Horstmann, 1965; Henquell et al., 2001; Carrol et al., 2006). Additionally, Echovirus strain 11 has been shown to possess genetic homology to CVB3 (Auvinen and Hyypiä, 1990; Dahllund et al., 1995; Huttunen et al., 1996). Elucidating mechanisms by which other enteroviruses, such as echoviruses, can impact iBECs may provide valuable insights in future studies.

CVB3 infection of iBECs highlights two potential routes of CVB3 traversal across the BBB. Firstly, we found that CVB3 infection causes perturbations of TJs which coincide with drastic reduction in observed TEER, all of which are signs of compromised barrier function. This is highlighted through the disruption of Occludin, Claudin-5, and ZO-1 in CVB3 infection, where increasing disruption of these proteins can be seen in a time dependent manner (**Supplemental Figure S3**). This is relevant as it has been demonstrated that the CVB3 receptor is a component of TJs between epithelial cells (Coyne et al., 2007). The same study states that in epithelial cells, depletion of Occludin limits cellular CVB entry (Coyne et al., 2007). Our work includes the tight barrier claudin, Claudin-5, to examine TJ dysfunction in endothelial cells. Therefore, there is a potential role of TJ proteins in infection of BECs. Future work needs to be done to determine if import into BECs is dependent on specific endothelial markers, or if Occludin plays the same role in CVB3 infection of endothelial cells (Dejana et al., 2009). Secondly, our transwell iBEC/HeLa data suggest that CVB3 may be able to cross the BBB despite the intact barrier integrity measured by TEER at short time points. We hypothesize that this is likely a byproduct of the infected BECs shedding infectious virus bilaterally, which in the context of the brain would suggest that circulating CVB3 would infect the BBB endothelium initially, and the infected cells would release virus toward the brain. In theory, shedding of virus into the brain from the infected BBB is likely how at least initial brain infection occurs; however future studies will assess if gradual BBB permeability may allow for pericellular movement of the virus.

The aspect of CVB3-mediated BBB permeability may have implications beyond potential avenues for brain infection. Loss of BBB function has been associated with a litany of neurological diseases including Alzheimer’s disease, amyotrophic lateral sclerosis, multiple sclerosis, stroke, epilepsy, meningitis, and many others (Van Vliet et al., 2007; Marchi et al., 2012; Winkler et al., 2013; Ortiz et al., 2014; Enzmann et al., 2018; Yang et al., 2019; Nehra et al., 2022; Pan and Nicolazzo, 2022; Spitzer et al., 2022). It is unclear if the infected BBB can eventually restore barrier properties, but if a potential persistent infection of the BBB could cause chronic barrier function loss, this could perhaps link even subclinical CVB3 infections with heightened susceptibility to late-onset diseases of the central nervous system.

## Data availability statement

The original contributions presented in the study are included in the article/**Supplementary material**. Further inquiries can be directed to the corresponding authors.

## Author contributions

JS, JM and BK conceived the study, designed the experiments, and wrote the manuscript. JS, JM, GH., EE, SS, and NV performed experiments and collected data. JS, BK, and JM analyzed the data. All authors contributed to the article and approved the submitted version.

## Supplementary material

Supplementary Figure 1 Characterization of the iBEC model. Immunostaining detecting endothelial markers PECAM (CD-31) and VE-Cadherin. Transporters Glut1 and P-glycoprotein (P-gp). TJs Claudin-5, Occludin, and Zona Occludin-1 (ZO-1). Scale bars = 50µm.

Supplementary Figure 2 Viability of iBECs stabilizes to maintain a live, intact barrier iBECs were infected with CVB3 at MOI 10 and assessed for cell viability at 48 h PI, 72 **h** PI, 5 days PI, and 9 days PI. Samples were taken of attached monolayer and counting with trypan blue counts of live cells expressed in percent is shown.

Supplementary Figure 3 iBECs have dramatically reduced Occludin localization at late infection timepoints. Immunostaining detecting Occludin localization **(A)**, Claudin-5 localization **(B)**, and ZO-1 localization **(C)** in iBECs either infected with eGFP-CVB3 at MOI 10 or mock treated with equivalent volume DMEM for 72 h. Immunostaining was conducted at 5 days PI for Occludin localization **(D)**, Claudin-5 localization **(E)**, and ZO-1 localization **(F)** in iBECs either infected with eGFP-CVB3 at MOI 10 or mock treated with equivalent volume DMEM for 5 days. Corresponding phase contrast images of the same field are shown below. Scale bars= 100 µm.

Supplementary Figure 4 Infected iBECs exhibit prolonged viral shedding. Daily plaque assays quantifications taken from tissue culture plastic coated wells of infectious viral titers in culture media from iBECs infected monolayers with eGFP-CVB3 at either MOI 1 or MOI 10. Crimson dotted line represents input viral inoculum for MOI 1 and black dotted line represents input viral inoculum for MOI 10 (Error bars represent standard deviation).

Supplementary Figure 5 TEER gradually declines in iBECs infected at MOI 1. iBECs were infected with eGFP-CVB3 at MOI 1 for the durations indicated. Transendothelial electrical resistance (TEER) in iBECs either infected with eGFP-CVB3 at MOI 1 or mock treated with equivalent volume DMEM. Relative TEER reading for virally-infected group was normalized to mock-infected group at each timepoint. (*p<0.05, **p<0.01, ***p<0.001; student’s *t*-test; n=3. Error bars represent standard deviation).

Supplementary Figure 6 Quantification of live virus across iBEC transwells. iBECs were seeded in transwells or compared with blank wells in cell media. iBECs or blank transwells were then infected with eGFP-CVB3 at MOI 10 (or equal viral on blank transwells) with the viral inoculum added into the top of the transwell. Plaque assay quantifications of infectious viral titers in culture media are shown from the top and bottom of transwells collected at 6 h PI **(A)**, 24 h PI **(B)**, and from cell lysates 24 h PI **(B)**. (n=3, n=2 for mock 48 h PI time point. Error bars represent standard deviation).
