# Supplementary figures and images for: Coxsackievirus B3 infects and disrupts human induced-pluripotent stem cell derived brain-like endothelial cells
Source: Front Cell Infect Microbiol. 2023 Apr 17;13:1171275. doi: 10.3389/fcimb.2023.1171275 (PMC10149843; doi:10.3389/fcimb.2023.1171275)

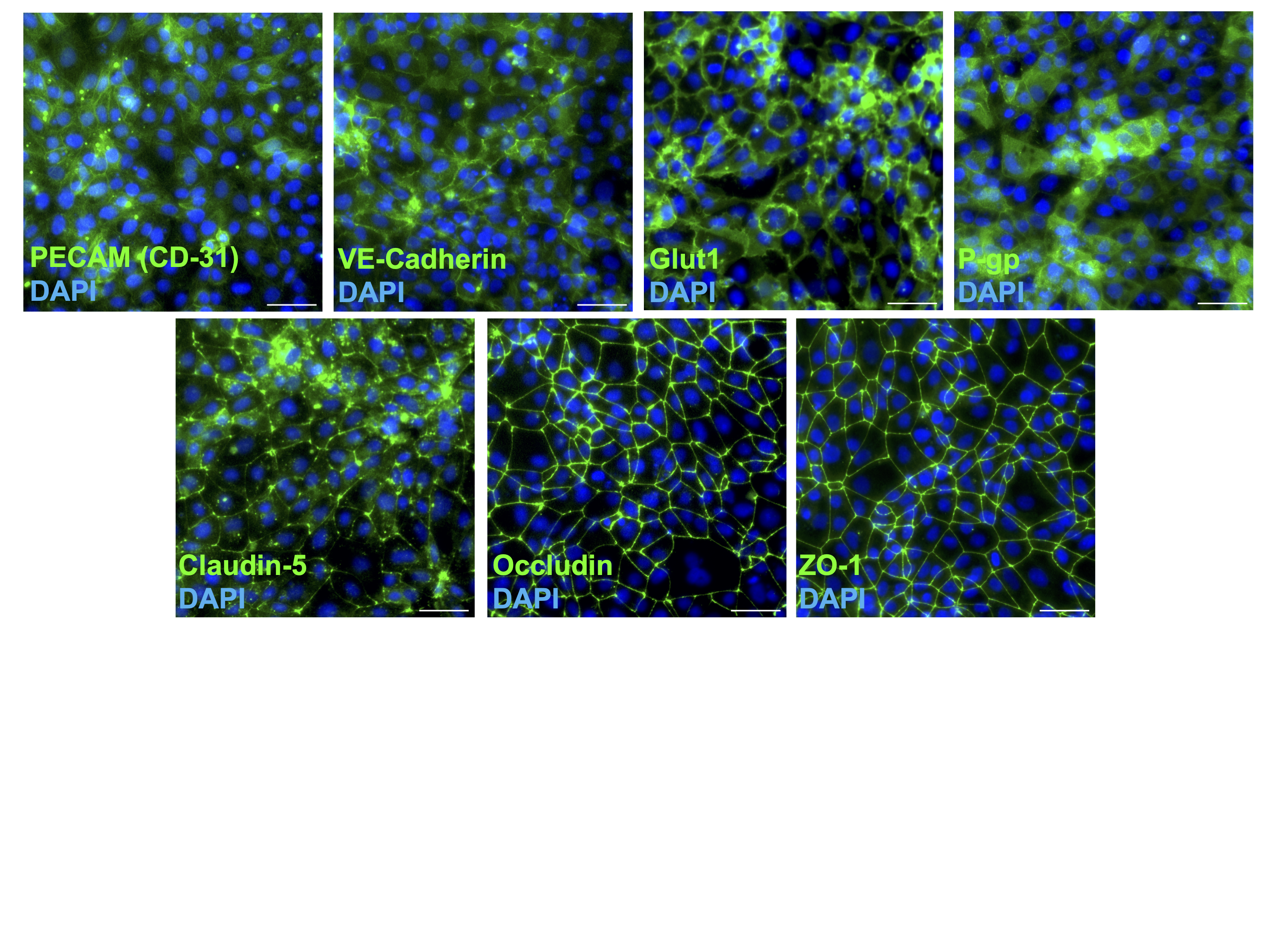

Supplement: Supplementary Figure 1 — Characterization of the iBEC model. Immunostaining detecting endothelial markers PECAM (CD-31) and VE-Cadherin. Transporters Glut1 and P-glycoprotein (P-gp). TJs Claudin-5, Occludin, and Zona Occludin-1 (ZO-1). Scale bars = 50µm. [file Image_1.tiff]

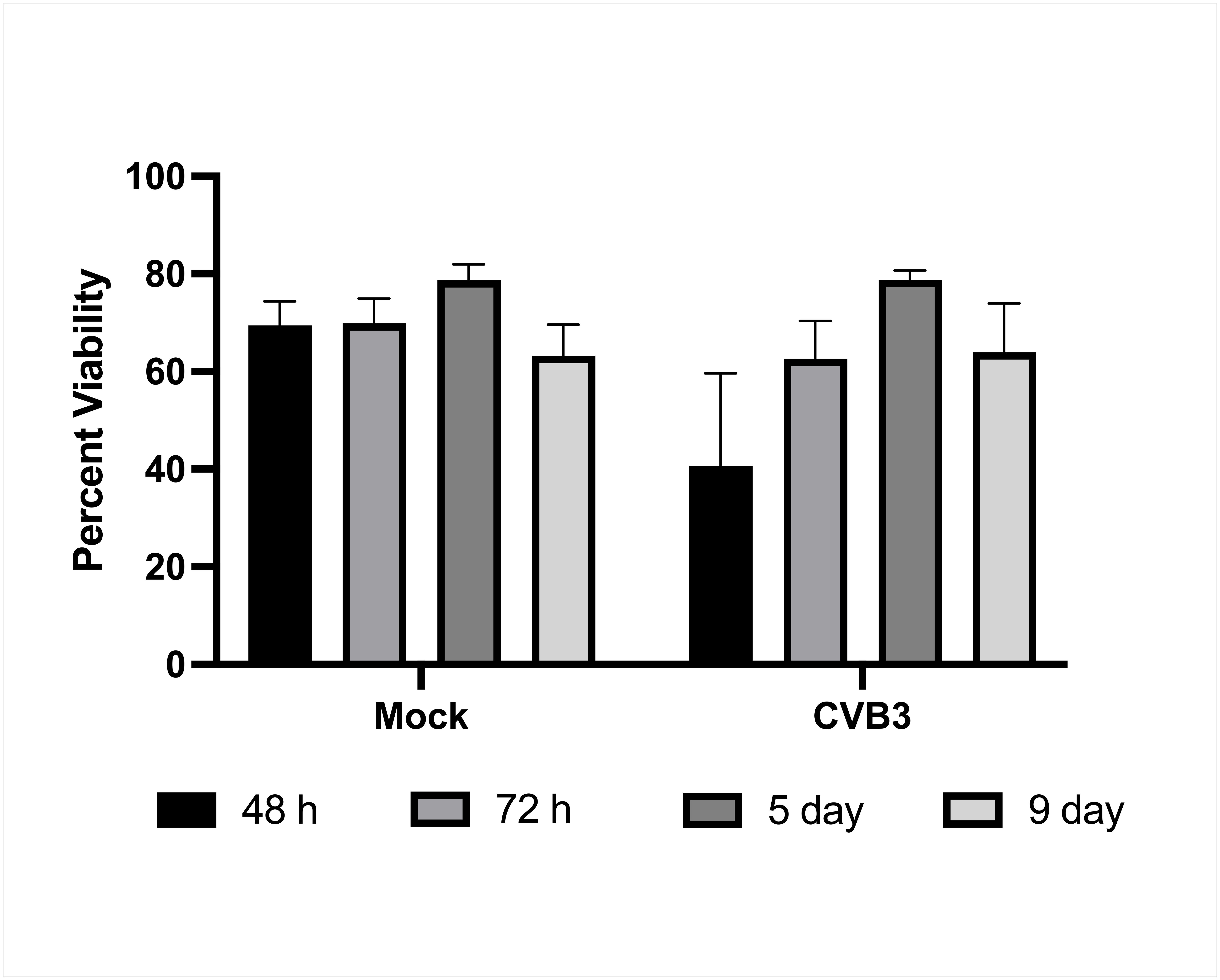

Supplement: Supplementary Figure 2 — Viability of iBECs stabilizes to maintain a live, intact barrier iBECs were infected with CVB3 at MOI 10 and assessed for cell viability at 48 h PI, 72 h PI, 5 days PI, and 9 days PI. Samples were taken of attached monolayer and counting with trypan blue counts of live cells expressed in percent is shown. [file Image_2.tiff]

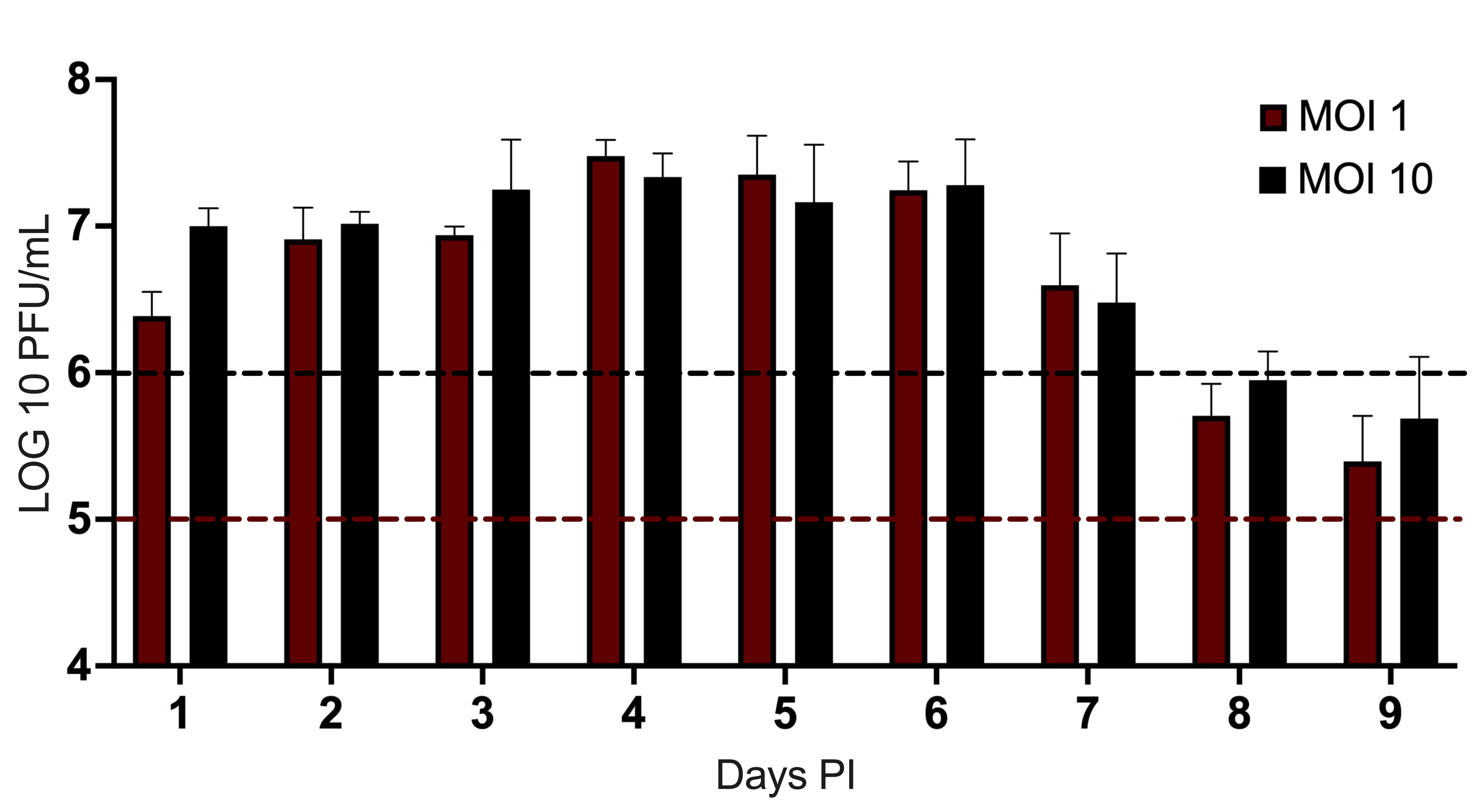

Supplement: Supplementary Figure 4 — Infected iBECs exhibit prolonged viral shedding. Daily plaque assays quantifications taken from tissue culture plastic coated wells of infectious viral titers in culture media from iBECs infected monolayers with eGFP-CVB3 at either MOI 1 or MOI 10. Crimson dotted line represents input viral inoculum for MOI 1 and black dotted line represents input viral inoculum for MOI 10 (Error bars represent standard deviation). [file Image_4.tiff]

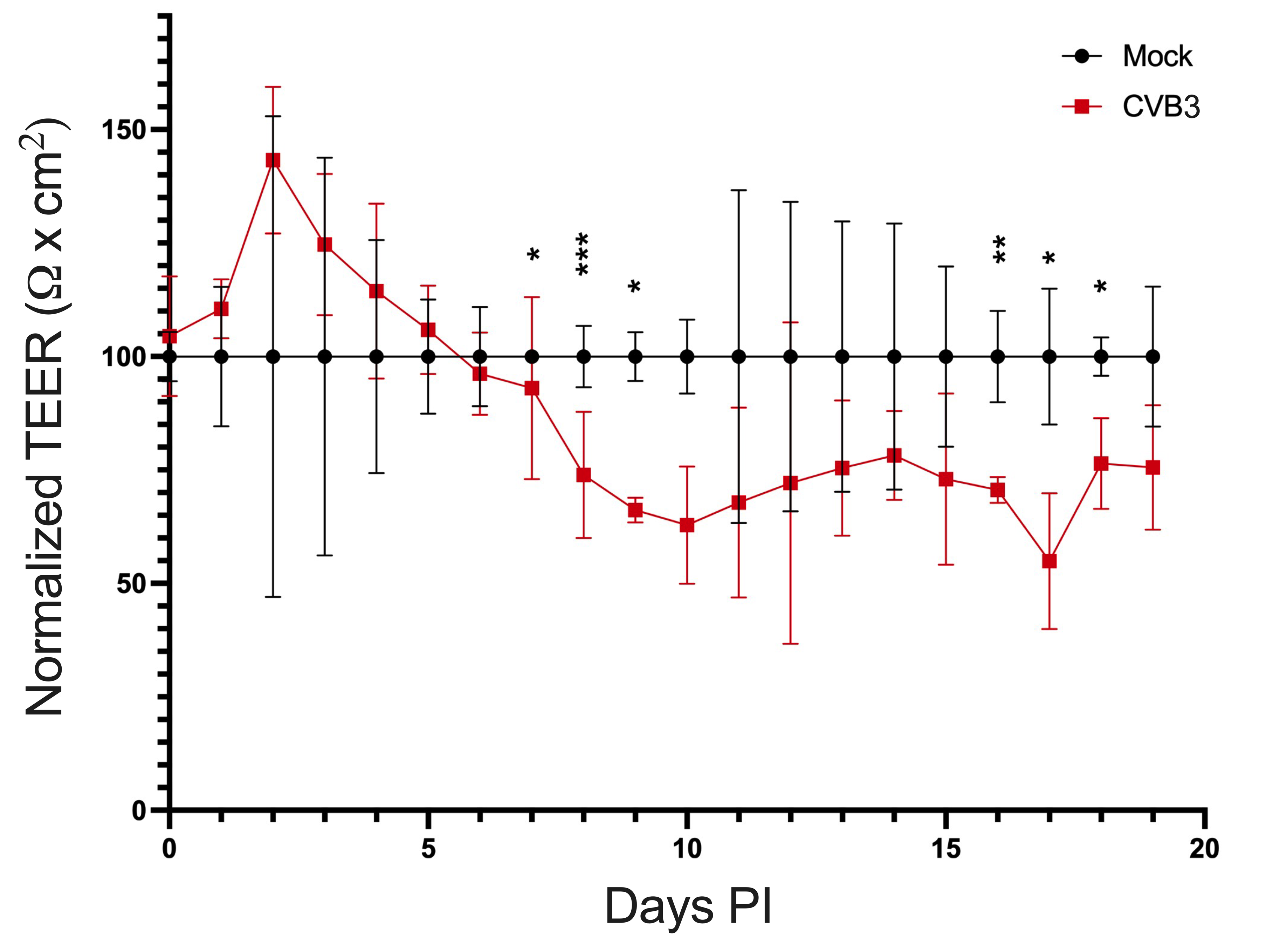

Supplement: Supplementary Figure 5 — TEER gradually declines in iBECs infected at MOI 1. iBECs were infected with eGFP-CVB3 at MOI 1 for the durations indicated. Transendothelial electrical resistance (TEER) in iBECs either infected with eGFP-CVB3 at MOI 1 or mock treated with equivalent volume DMEM. Relative TEER reading for virally-infected group was normalized to mock-infected group at each timepoint. (*p<0.05, **p<0.01, ***p<0.001; student’s t-test; n=3. Error bars represent standard deviation). [file Image_5.tiff]

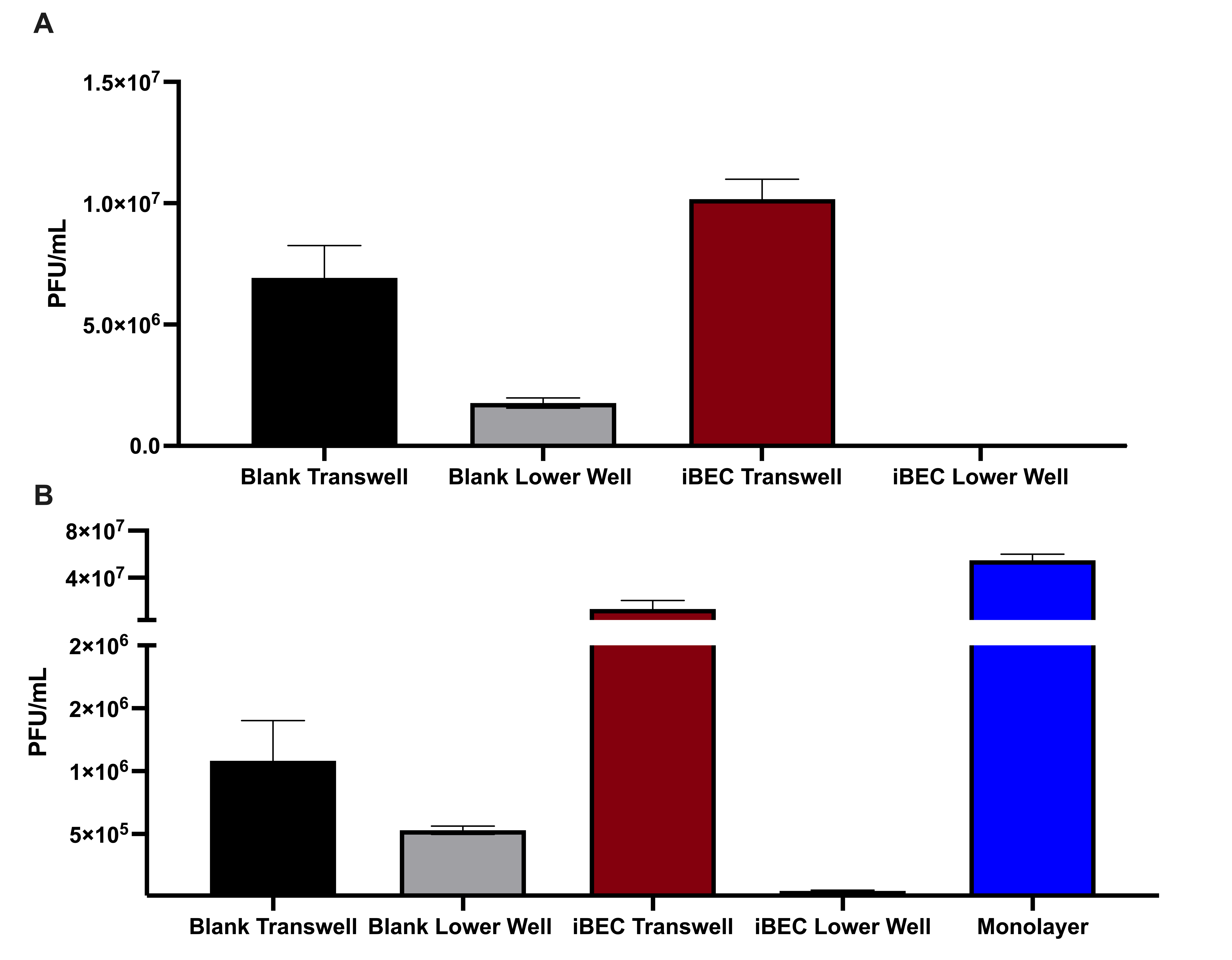

Supplement: Supplementary Figure 6 — Quantification of live virus across iBEC transwells. iBECs were seeded in transwells or compared with blank wells in cell media. iBECs or blank transwells were then infected with eGFP-CVB3 at MOI 10 (or equal viral on blank transwells) with the viral inoculum added into the top of the transwell. Plaque assay quantifications of infectious viral titers in culture media are shown from the top and bottom of transwells collected at 6 h PI (A), 24 h PI (B), and from cell lysates 24 h PI (B). (n=3, n=2 for mock 48 h PI time point. Error bars represent standard deviation). [file Image_6.tiff]
